# Supplementary material for: Hepatitis B vaccine uptake and associated factors among adults of Gulu city in Northern Uganda: A community-based cross-sectional study
Source: PLoS One. 2024 Mar 1;19(3):e0297617. doi: 10.1371/journal.pone.0297617 (PMC10906836; doi:10.1371/journal.pone.0297617)
Supplement: Attachment — Submitted filename: Response to Reviewers.docx [file pone.0297617.s001.docx]

PONE-D-23-29648

Hepatitis B vaccine uptake and associated factors among adults of Gulu City in Northern Uganda: A community-based cross-sectional study.

Dear Dr. Jason T. Blackard,

Academic Editor

PLOS ONE

We thank you for handling this manuscript and for the useful review comments that aimed to improve the quality of this manuscript. We have revised the manuscript as per the editor and reviewers’ comments and here are our point-by-point responses to the comments.

Editor Comments:

This is a cross-sectional, community-based study of HBV vaccination in Northern Uganda.

Overall, the results of this study are not surprising and the writing would benefit from review by a native English speaker and/or a professional editing service.

Authors’ response: Thank you, the manuscript has been reviewed for grammar and punctuations.

Were number of HBV vaccine doses received self-reported by the study participants or extracted from medical records?

Authors’ response: Thank you. The number of HBV vaccine doses received were self-reported by the participants. We acknowledged this in the study limitation.

Were HBV antibodies measured / quantified to know if the vaccination series worked effectively?

Authors’ response: Good question. The HBV antibodies were not measured / quantified. This was out of the scope of our study which aimed at evaluating self-reported uptake of Hepatitis B vaccine. Perhaps this could be an area for further studies.

It is not clear how knowledge of HBV transmission routes and/or health education were provided in the first place.  Were these part of the study design or provided in some other setting?

Authors’ response: Both knowledge of HBV transmission routes and health education were asked in the questionnaire.

Knowledge of HBV transmission routes was assessed using two categories. Participants who stated at least one correct method of transmission was categorized as “knowledgeable” while those who didn’t know any route of transmission and those who wrongly stated all the transmission routes were categorized as “not knowledgeable”. Health education was assessed using two categories, “yes” or “no”. the participants were asked whether they had received health education from health workers. Those who answered ‘yes’ were considered health educated and those who answered ‘no’ were considered not health educated. All of them were part of the study design.

COMMENTS TO REVIEWERS

Reviewer #1: Title: This is misleading as Gulu city is not equivalent to Northern Uganda. it should read" Gulu City in Northern Uganda"
Methods: There should be more description or details of the proportional sampling of the parishes and systematic random sampling of households. What time of the day were questionnaires administered as the time of day can also determine the types of respondents.

Authors’ response: We agree with the reviewer. The title was amended reading ‘Hepatitis B vaccine uptake and associated factors among adults of Gulu City in Northern Uganda. A community based cross sectional study.

Methods: A total of 16 of the 32 (50%) parishes in Gulu City were randomly sampled using number tables. Stratified random sampling proportionate to size was done to determine the number of participants from each parish.

Random samples were taken from each parish in proportion to the population in that parish. We used the proportionate stratified random sampling formula: nh = (Nh / N) *n where nh =sample size for each Parish, Nh =population size in each parish, N =size of the entire population in Gulu City, n = size of the entire sample.

Systematic random sampling: we calculated the sampling interval by dividing the entire population size of that parish by the desired sample size for that parish. Sampling of the households began from anywhere selected at random.

Questionnaires were administered from 8:00am to 6:00pm from Monday to Saturday.

Results:
figure 2-No need for 2 bars i.e., freq/percentage. You can use percentages and put the figure in brackets on top of the bar.

Authors’ response: I have removed 1bar and for data label, I have used percentages and figures in brackets.

Figure 3: No need for a bar chart. the data can be presented in a table and it will be clear.

Authors’ response: Thank you for the suggestion, we have removed the bar chart and incorporated it in figure two. Its contents have been put as a caption under figure two.
Table 2: Multivariable analysis-The table can be improved by including only variables that had significant p values in the univariate analysis
No need to write AOR in full in results. Use of times ie 4 times makes sentence clearer in this case as opposed to 4 folds

Authors’ response: table 2 was modified. Only variables that had significant p values were included.

The words adjusted odds ratio were removed.

The use of 4 folds were eliminated and replaced by 4 times.
Discussion: There is quite an amount of repetition of results in this section. This should be addressed and reduced. The discussion could also be beefed up.

Authors’ response: We agree with the reviewer. The discussion section was edited.

Limitations: Another limitation of the study is the time of conducting the survey as this could affect the category of respondents available and present in the various households.

Authors’ response: Thank you, this was included under limitations.

General: There are grammatical errors which should be corrected. Hepatitis B, the B should be in capitals

Authors’ response: Grammatical errors were corrected. The B in Hepatitis B has been capitalised throughout the manuscript.

Reviewer #2: The manuscript is a basic public health study. The study should have also explained Uganda's policy on HBV vaccination policy on infants and adults within the background section. This should then be linked with adult population factors contributing to failure to get vaccinated.

Authors’ response: Thank you, we agree with the reviewer and have added a substantial amount of information in the introduction. Uganda policy on hepatitis B prevention, care and treatment has been included in the background section. A few factors contributing to low vaccine uptake have also been included in the background.

Table 1: how many of these study participants were healthcare workers? Table 1: Level of income, what currency is. Is there any difference between children population and adult regarding vaccination status? While this study may give an impression of significance, unfortunately its more applicable to Uganda policy makers, and more relevant there. It does not really add new info to current knowledge of HBV vaccination

Authors’ response: Good insight. We are not sure how many of the study participants were healthcare workers. The study was done in households within the community and definitely some of the participants might have been healthcare workers.

Table 1: under level of income, the currency used was Uganda shillings. I have indicated it in table 1 and a conversion of USD to UGX has been indicated.

Regarding HBV vaccination status, in Uganda, children born starting from 2002 onwards received the pentavalent vaccine DPT which has a combination of DPT, Hep B, HCV. Individuals born before 2002 were not vaccinated against HBV.
